# Fetal Growth Is Associated with Maternal Fasting Plasma Glucose at First Prenatal Visit

**DOI:** 10.1371/journal.pone.0116352

**Published:** 2014-12-31

**Authors:** Bin Liu, Haitian Chen, Yun Xu, Chongyou An, Lieqiang Zhong, Xiaohui Wang, Ying Zhang, Hanqing Chen, Jinxin Zhang, Zilian Wang

**Affiliations:** 1 Department of Obstetrics and Gynecology, The First Affiliated Hospital of Sun Yat-sen University, Guangzhou, PR China; 2 Department of Endocrinology, The Sixth Affiliated Hospital of Sun Yat-sen University, Guangzhou, PR China; 3 Department of Medical Statistic and Epidemiology, School of Public Health, Sun Yat-sen University, Guangzhou, PR China; University of Missouri, United States of America

## Abstract

**Aims:**

Fasting plasma glucose (FPG) concentration measured at the first prenatal visit is a predictor of gestational diabetes mellitus (GDM); however, whether this test is indicative of fetal growth has not been clarified. Thus, the purpose of this study was to determine whether birth weight and birth length were related to FPG levels at the first prenatal visit.

**Materials and Methods:**

Research samples were collected from pregnant women who took an FPG test at their first prenatal visit (10–24 gestational weeks), received regular prenatal care, and delivered in our center. FPG value, maternal pre-gravid BMI, weight gain before FPG test, before and after Oral Glucose Tolerance Test (OGTT), neonatal birthweight, birth length, Ponderal Index and birthing method were recorded for analysis. Data were analyzed by independent sample *t* test, Pearson correlation, and Chi-square test, followed by partial correlation or logistic regression to confirm differences. Statistical significance level was α = 0.05.

**Results:**

2284 pregnant women, including 462 GDM and 1822 with normal glucose tolerance (NGT) were recruited for the present study. FPG concentration at the first prenatal visit was associated with neonatal birth weight (partial correlation coefficient *r′* = 0.089, *P*<0.001) and birth length (partial correlation coefficient *r′* = 0.061, *P* = 0.005), but not with Ponderal Index or birthing method. Maternal pre-gravid BMI was associated with FPG value (partial correlation coefficient *r′* = 0.113, P<0.001). FPG concentration at the first prenatal visit (OR = 2.945, P<0.001), weight gain before OGTT test (OR = 1.039, P = 0.010), and age (OR = 1.107, P<0.001) were independent related factors of GDM.

**Conclusion:**

Fasting plasma glucose concentration at the first prenatal visit is associated with fetal growth. Maternal pre-gravid BMI and weight gain are related to glucose metabolism.

## Introduction

Gestational diabetes mellitus (GDM) is a common complication with increasing world-wide prevalence [1], [2]. A large number of adverse fetal consequences, including macrosomia, respiratory distress syndrome, and hypoglycaemia, are related to GDM [3]. Moreover, offspring of GDM mothers are more likely to develop diabetes [4], obesity [4]–[6], and metabolic disorder [7] in child- and adulthood.

Recently, Zhu *et al* [1] reported that hyperglycemia at the first prenatal visit is an early indicator of subsequent GDM. Their study demonstrated the importance of an fasting plasma glucose (FPG) test during the early stages of pregnancy. In the present study, we aim to verify the significance of the FPG test in two ways: first, based on the Developmental Origins of Health and Disease (DOHaD) theory [8], [9], maternal metabolism influences intrauterine environment, and poor intrauterine nutritional experiences may affect fetal growth, leading to adverse consequences for the offspring [6], [10], [11]. So, the first purpose of the present study is to investigate the relationship between FPG concentration in the early stages of pregnancy and fetal growth. Second, when analyzing the impact of FPG levels on GDM diagnosis, the article by Zhu *et al* [1] did not take maternal BMI and weight change into consideration. Pre-gravid weight and weight gain heavily influences maternal metabolism during pregnancy [12]–[14], and some recent studies [15]–[17] identified pre-pregnancy obesity/overweight and excessive gestational weight gain as risk factors of gestational diabetes and macrosomia. Therefore, we also set out to examine possible correlations between maternal BMI, weight gain, and glycemic metabolism.

## Materials and Methods

### Study population

Singleton pregnant women who took an FPG test during the first prenatal visit (10–24 gestational weeks), received regular prenatal care, and delivered in our center between March 2011 and December 2012 were recruited for the study population. The present study has been approved by the ethical committees of the The First Affiliated Hospital of Sun Yat-sen University, and all participants provided written informed consent.

### Data collection

During the first prenatal visit, fasting plasma glucose levels were tested using venous plasma obtained after at least 8 h of fasting. A 75-g Oral Glucose Tolerance Test (OGTT) was performed between 24 to 28 gestational weeks. Maternal clinical data included age, gravity, parity, height and gestational age. Maternal weights before pregnancy, at the first prenatal visit, at the time of OGTT test, and just prior to delivery were recorded to monitor gestational weight gain. Development of gestational diabetes mellitus, neonatal birth weight, birth length, Ponderal index [PI = mass(kg)/height(m)^3^], and birthing method (vaginal vs. cesarean) were taken as major outcomes.

### Statistical analysis

Data was analyzed using SPSS Version 17.0. Continuous and normally distributed variables were described as mean± SD and analyzed by independent sample *t* test or Pearson correlation. Categorical variables were described as proportions and examined with Chi-square test. Factors of interest were further studied using partial correlation analysis or logistic regression analysis to confirm significance.

## Results

### Baseline characteristics of the study population

**Figure 1:**
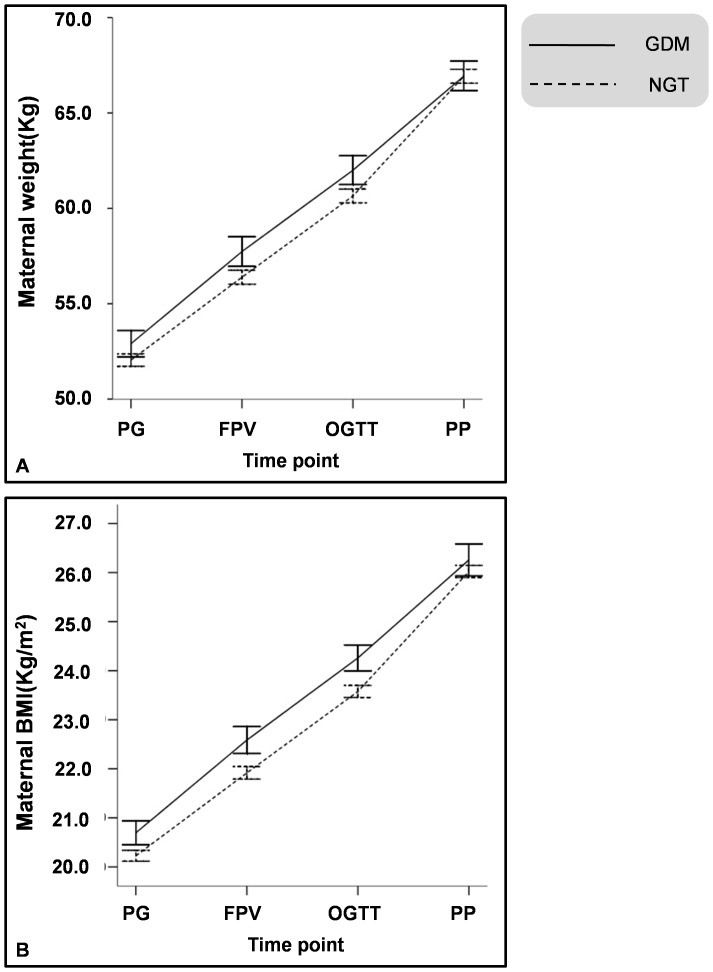
Weight and BMI changes in pregnant women complicated with GDM and control (NGT) groups during pregnancy. Panel A. Weight and weight gain in GDM and NGT groups before pregnancy (Pregravid, PG), at first prenatal visit (FPV), at OGTT time (OGTT), and before delivery (Prepartum, PP). Panel B. BMI of GDM and NGT group before pregnancy (Pregravid, PG), at first prenatal visit (FPV), at OGTT time (OGTT), and before delivery (Prepartum, PP).

According to inclusive and exclusive criteria, a total of 2284 pregnant women were recruited for this study. Among them, 462 developed gestational diabetes mellitus and 1822 were of normal glucose tolerance. Pregnant women in the GDM group had higher weight and BMI before pregnancy (Table 1), more weight gain before FPG test (4.83±4.15 vs. 4.34±3.96, P = 0.020) and OGTT test (9.09±3.60 vs 8.60±3.46, P = 0.007), but had less weight gain after OGTT test (4.95±3.29 vs. 6.27±2.99, P<0.001) (Fig. 1).Subsequently, there was no statistically significant weight difference before delivery between the two groups (Table 1).

**Table 1:** Baseline characteristics of the research population.

	GDM	NGT	P
Number	462	2284	
Age(means ± SD)	31.3±4.1	29.5±3.7	<0.001
Parity			0.271
Nullipara (%)	391 (84.6)	1578 (86.6)	
Multipara (%)	71 (15.4)	244 (13.4)	
Gestational age (Day)			
At first prenatal visit	122.5±32.4	123.9±29.5	0.372
At OGTT test	189.0±28.9	185.1±19.0	0.006
At delivery	271.4±10.4	273.5±12.9	0.001
Maternal weight (kg)			
Before pregnancy	52.90±7.50	52.05±7.15	0.022
At first prenatal visit	57.77±8.50	56.39±8.01	0.002
At OGTT test	62.00±8.24	60.65±7.94	0.002
At delivery	66.94±8.47	66.93±7.94	0.979
Maternal height (cm)	159.8±4.6	160.3±4.8	0.036
Maternal BMI (kg/m^2^)			
Before pregnancy	20.69±2.67	20.23±2.42	0.001
At first prenatal visit	22.59±3.00	21.92±2.76	<0.001
At OGTT test	24.26±2.89	23.58±2.65	<0.001
At delivery	26.26±3.57	26.02±2.66	0.115
Fasting plasma glucose	4.45±0.45	4.27±0.37	<0.001
Neonatal gender			0.434
Male (%)	233(50.4)	956(52.5)	
Female (%)	229(49.6)	866(47.5)	
Birth weight (g)	3147±486	3162±439	0.561
Birth Length (cm)	49.4±2.1	49.5±2.0	0.149
Birth method			0.001
Vaginal (%)	185 (40.0)	886 (48.6)	
Cesarean (%)	277 (60.0)	936 (51.4)	

**Figure 2:**
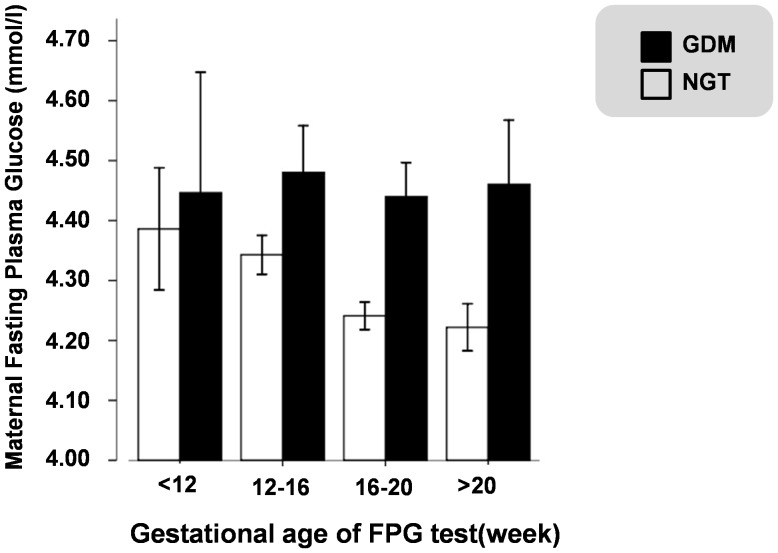
Fasting plasma glucose concentrations for GDM and NGT groups in different gestational weeks.

Fasting plasma glucose concentration in the GDM group was higher than the NGT group (Table 1).Considering FPG levels may fluctuate according to gestational week, we analyzed this difference in subgroups (<12 weeks, 12–16 weeks, 16–20 weeks, 20–24 weeks). Results demonstrated that GDM mothers had significantly higher FPG levels in all subgroups except in the <12 week group, indicating that the relationship between FPG and GDM appears during the second trimester (Fig. 2).

### Maternal FPG concentration and neonatal birthweight, birth length, Ponderal index, and birthing method

FPG concentration was associated with neonatal birth weight (partial correlation coefficient *r′* = 0.089, P<0.001), after adjusting for maternal age, pre-gravid BMI, weight gain before and after OGTT, gestational age, GDM and neonatal gender (Table 2). This association is more pronounced in male (partial correlation coefficient *r′* = 0.103, P<0.001) than in female (partial correlation coefficient *r′* = 0.070, P = 0.021) infants, and in GDM (partial correlation coefficient *r′* = 0.158, P = 0.001) than in NGT (partial correlation coefficient *r′* = 0.065, P = 0.006) pregnancies.

**Table 2:** Relationship between maternal glycemic metabolism, maternal weight pattern and neonatal birthweight, birth length and Ponderal index.

	Birth weight			Birth length			Ponderal index		
	r	r′	P for r′	r	r′	P for r′	r	r′	P for r′
Maternal age	0.022	0.067	0.001	−0.005	0.044	0.043	0.032	0.029	0.182
Gestational age	0.524	0.546	<0.001	0.498	0.511	<0.001	0.131	0.132	<0.001
Maternal BMI (kg/m^2^) Before pregnancy	0.149	0.185	<0.001	0.053	0.063	0.004	0.130	0.129	<0.001
Maternal weight gain(kg)									
Weight gain Before OGTT test	0.097	0.160	<0.001	0.072	0.101	<0.001	0.330	0.033	0.128
Weight gain between OGTT and delivery	0.079	0.082	<0.001	0.051	0.028	0.208	0.028	0.033	0.138
Fasting plasma glucose (mmol/L)	0.073	0.089	<0.001	0.041	0.061	0.005	0.042	0.038	0.083

Partial correlation between FPG concentration and birth weight, birth length and Ponderal index were adjusted with maternal age, pre-gravid BMI, weight gain before and after OGTT, gestational age, GDM diagnosis and neonatal gender. r: correlation coefficient; r′: partial correlation coefficient.

Interestingly, although birth weight was not significantly different when comparing the GDM and NGT groups (Table 1), fasting glucose levels measured by OGTT test was correlated with neonatal birthweight (partial correlation coefficient *r′* = 0.111, P<0.001), after adjusting for maternal age, pre-gravid BMI, weight gain before and after OGTT, gestational age, GDM and neonate gender. However, OGTTs 1 and 2 hours post-meal did not have significant correlations with birth weight. These results indicate that fetal growth is mostly controlled by maternal basal glycemic levels.

Although a bivariate correlation test between FPG and neonatal birth length was not statistically significant, a further partial correlation confirmed this relationship (partial correlation coefficient *r′* = 0.061, P = 0.005), after adjusting for maternal age, pre-gravid BMI, weight gain before and after OGTT, gestational age, GDM and neonatal gender (Table 2).

Further, we investigated the relationship between maternal fasting glucose and neonatal Ponderal Index (PI). FPG concentration at first prenatal visit was not related to neonatal PI, in neither Pearson correlation nor in partial correlation tests (Table 2). However, the fasting glucose value in OGTT test was associated with neonatal Ponderal index (partial correlation coefficient *r′* = 0.080, P<0.001), after adjusting for maternal age, pre-gravid BMI, weight gain before and after OGTT, gestational age, GDM and neonatal gender.

Although FPG value in the vaginal birth group was lower than cesarean group in independent student t test (FPG: 4.28±0.38 vs. 4.33±0.40, P = 0.011), this difference failed to be confirmed by logistic regression, after adjusting for maternal age, pre-gravid BMI, weight gain before and after OGTT, gestational age, and GDM.

### Maternal pre-gravid BMI and weight gain is associated with glycemic metabolism

**Figure 3:**
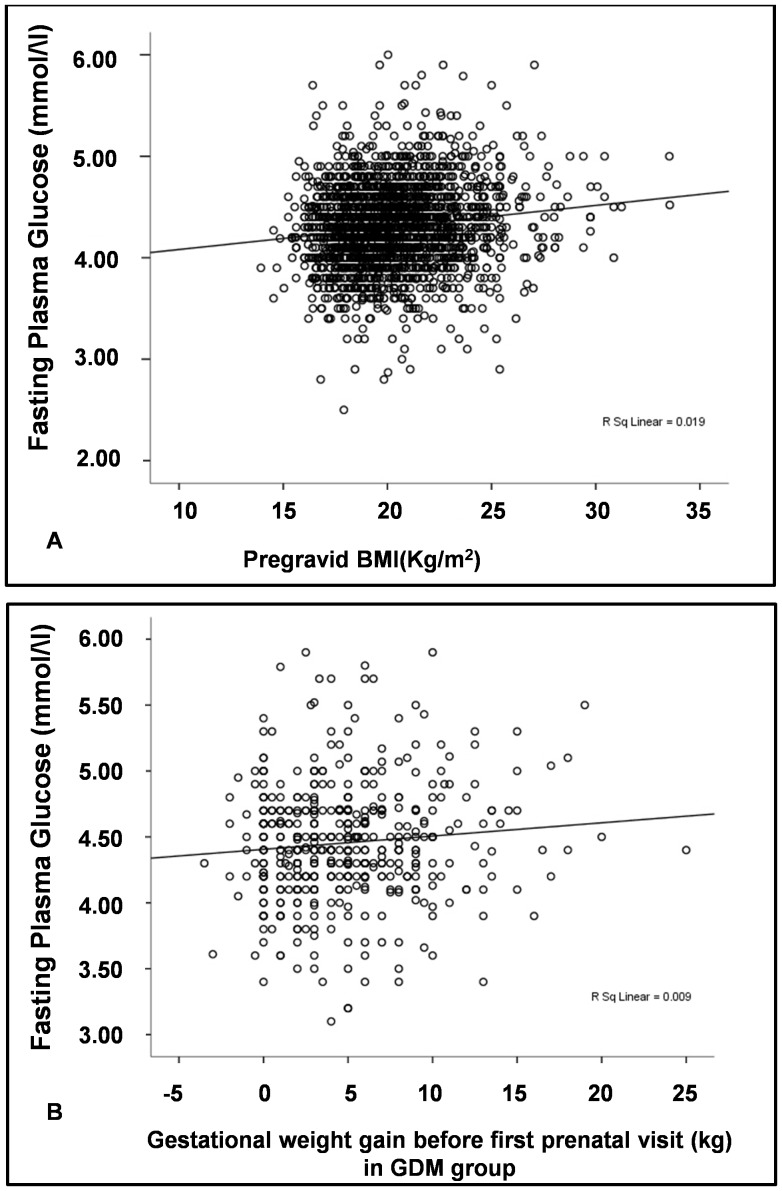
Relationship between fasting plasma glucose concentration, pre-gravid BMI and weight gain before FPG test. Panel A. FPG is associated with pre-gravid BMI across the tested population. Panel B. FPG is associated with weight gain before FPG test in GDM group.

As shown in Fig. 3A, FPG levels are associated with pre-gravid BMI across the whole population (partial correlation coefficient *r′* = 0.113, P<0.001), after adjusting for age and weight gain before FPG. Gestational weight gain before FPG test is related to FPG levels in those who developed GDM later (partial correlation coefficient *r′* = 0.099, P = 0.033) (Fig. 3 B) but not across the whole study population (partial correlation coefficient *r′* = 0.017, P = 0.430) nor in the NGT group (partial correlation coefficient *r′* = −0.021, P = 0.366).

Additionally, we conducted a logistic regression to investigate the relationship of FPG, pre-gravid BMI, and weight gain before OGTT test with GDM. Results showed that FPG levels in first prenatal visit (OR = 2.945, P<0.001), weight gain before OGTT test (OR = 1.039, P = 0.010), and age (OR = 1.107, P<0.001) were statistically significant predictors of GDM.

## Discussion

Gestational diabetes mellitus is a common disease in pregnant women all over the world [2]. A recent study has verified that basal glucose levels during early gestational period is related to the development of GDM [1]. Thus, we further investigated whether basal glucose levels were associated with pregnancy outcome. The main findings of the present article is that basal glucose concentration (at first prenatal visit and at OGTT test) is associated with neonatal birth weight and length, while maternal pre-gravid BMI and gestational weight gain is related to glycemic metabolism.

According to the well-known Pederson hypothesis [18], maternal hyperglycemia induces fetal overgrowth *in utero*. Most often in the clinic, maternal glucose management begins after GDM diagnosis (after the OGTT test at 24–28 gestational weeks), but the influence of hyperglycemia on fetal overgrowth can begin earlier than this test [19]. Our data demonstrates that even prior to the OGTT test, maternal fasting glucose concentration is correlated with neonatal birthweight. This result is consistent with previous reports in other populations [20], [21] but also shows that the trend is present in a broader range of gestational weeks. Our data also demonstrates that FPG is associated with birth length. Taken together, this evidence demonstrates the importance of the FPG test in the early stages of pregnancy, not only as an indicator of GDM, but also a predictor of fetal growth.

Interestingly, neonatal birth weight is also correlated with fasting glucose concentration measured by OGTT test, but not with glycemic levels 1 or 2 hours post-meal. Additionally, in the present population, newborns of GDM mothers did not have higher birthweight compared to those of NGT mothers, even taking into consideration that GDM mothers gained less weight after the OGTT test. These results indicated that maternal fasting glycemia, rather than after-meal glycemia or GDM diagnosis, strongly affects fetal growth. Thus, management of fasting glucose levels during the entire gestational period maybe an effective way to control fetal overgrowth.

Ponderal Index (PI) is an indicator of fetal growth status. We applied PI to investigate the relationship between fetal growth status and FPG. Results demonstrated that neonatal birth PI was not related to FPG at the first prenatal visit, but was associated with the FPG levels at the time of the OGTT test. These results indicated that neonatal PI would not be affected by maternal basal glycemia until mid and late gestation. Thus, a single FPG test at the first prenatal visit would provide limited information on Ponderal Index at birth.

Since maternal obesity and excessive gestational weight gain are risk factors of GDM [22], we further investigated how pre-gravid BMI and weight gain impact glucose metabolism. We found that FPG concentration was positively associated with pre-gravid BMI and weight gain before FPG test (in GDM group); and weight gain before OGTT test was correlated with GDM diagnosis. These findings were consistent with a recent study [23], and indicated that management of pre-gravid weight and gestational weight can play a critical role in preventing GDM development. However, since the correlation between pre-gravid BMI and FPG value is mild, it is probable there are other powerful causative variables that affect FPG. Thus, further studies containing more co-factors are needed.

Maternal weight and weight gain also impacts fetal growth [15]–[17], [21]. However, in clinical practice, a women that is diagnosed as GDM is more likely to control her weight after OGTT test; therefore, in the present study, we also investigated how gestational weight gain before and after OGTT test influences GDM diagnosis and neonatal birthweight and birth length. The result showed that GDM mothers gained more weight before and less weight after OGTT test compared to the NGT group. This phenomenon illustrates that weight gain before OGTT test is associated with GDM diagnosis, while less weight gain after GDM diagnosis may contribute to prenatal weight and glucose management.

Weight gain before and after OGTT test are both related to neonatal birthweight, while neonatal birth length was related to maternal weight gain before, but not after OGTT test (Table 2). Therefore, weight gain control after conception should help contribute to fetal weight gain control, either directly or indirectly via basal glucose levels, while weight gain control after OGTT did not influence neonatal birth length.

In conclusion, our study demonstrates that maternal FPG concentration, pre-gravid BMI and gestational weight gain before and after OGTT test are associated with neonatal birth weight and length (excluding weight gain after OGTT test). Maternal weight and weight gain are related to glucose metabolism.
